# A Novel 9‐Channel ^1^H, 3‐Channel ^31^P Radiofrequency Coil for Interleaved Multinuclear Studies of Human Calf Muscle at 7 T

**DOI:** 10.1002/mrm.70249

**Published:** 2026-01-04

**Authors:** Veronika Cap, Vasco Rafael Rocha dos Santos, Kostiantyn Repnin, Onisim Soanca, Elmar Laistler, Peter Wolf, Graham J. Kemp, Roberta Frass‐Kriegl, Martin Meyerspeer

**Affiliations:** ^1^ High Field MR Center Center for Medical Physics and Biomedical Engineering, Medical University of Vienna Vienna Austria; ^2^ Division of Endocrinology and Metabolism, Department of Medicine III Medical University of Vienna Vienna Austria; ^3^ Department of Musculoskeletal and Ageing Science and Liverpool Magnetic Resonance Imaging Centre (LiMRIC) University of Liverpool Liverpool UK

**Keywords:** magnetic resonance imaging, magnetic resonance spectroscopy, muscle physiology, radiofrequency coil, ultra‐high field, X‐nucleus

## Abstract

**Purpose:**

To develop a double‐tuned calf coil with excellent ^1^H and ^31^P performance to study muscle physiology by interleaved ^1^H/^31^P MRS and parallel imaging at 7 T.

**Methods:**

The coil combines three ^1^H transceive dipoles, six ^1^H receive‐only loops and three ^31^P transceive loops, arranged in a nested, half‐cylindrical layout. Several different decoupling mechanisms were implemented to limit electromagnetic interactions within the ^1^H and ^31^P arrays, between transmit and receive elements as well as ^1^H‐^31^P cross‐coupling. A custom housing was designed for mechanical stability and ease of use. The ^1^H and ^31^P performance were investigated on phantoms and demonstrated in vivo in an exemplary measurement with interleaved, localized ^1^H/^31^P MRS in the gastrocnemius muscle of a healthy subject at rest, during exercise and recovery.

**Results:**

On phantoms, compared to a double‐tuned reference coil, the new coil showed 2.8‐fold higher ^1^H SNR, a stronger and more homogenous ^1^H transmit field, and 27% lower ^31^P SNR. It enabled four‐fold GRAPPA acceleration. In vivo the CH_2_ resonance of creatine and PCr were fitted from the unaveraged ^1^H and ^31^P spectra, which resulted in time courses with closely matching depletion and recovery constants.

**Conclusion:**

These results highlight the coil's suitability for the target application of advanced metabolic calf muscle studies. Its high ^1^H performance will enable ^1^H MRS acquisitions with improved spatial and temporal resolution and enable the implementation of MRS sequences that had previously been limited by insufficient SNR, which will generate new insights into muscle physiology.

## 1 Introduction

Magnetic resonance spectroscopy has been widely used for the non‐invasive study of muscle metabolism in several organs and tissues, including skeletal muscle. In particular, phosphorus (^31^P) MRS [1] offers unique access to key cytosolic metabolites of energy metabolism in working muscle: direct detection of adenosine triphosphate (ATP), phosphocreatine (PCr) and inorganic phosphate (Pi); measurement of cytosolic pH based on analysis of the Pi‐PCr chemical shift difference; and indirect estimation of the free cytosolic concentrations of Mg^2+^, adenosine diphosphate (ADP) and adenosine monophosphate (AMP). When the muscle is challenged in an exercise protocol, the kinetics of key metabolite measurements yields information about ATP turnover rate (and thus contractile efficiency), oxidative ATP production and ‘mitochondrial capacity’ (in lower intensity exercise and during post‐exercise recovery from exercise [2]), and (at higher exercise intensities) glycolytic ATP production [1]. This last involves net generation and accumulation of lactate, which is also directly detectable via ^1^H MRS [2], but requires complex spectral editing sequences to suppress overlapping lipid signals [3]. Acetyl carnitine, carnosine, taurine, lipids and creatine can also be studied through ^1^H MRS. Interestingly, the creatine CH_2_ resonance (Cr2) has been reported to fall during and recover post‐exercise, similar to PCr, while the CH_3_ resonance remained constant [4, 5]. MR imaging can be used to measure fat‐fraction [6, 7], perfusion via arterial spin labelling [8], and muscle fiber orientation via diffusion tensor imaging [9]; this last is relevant for MRS, because it affects the spectral appearance of extramyocellular lipids, and the coupling constants and visibility of Cr2 and lactate.

Combining ^31^P MRS, ^1^H MRS and advanced imaging protocols offers more comprehensive insight into muscle physiology by exploiting the complementary information each method supplies. Both ^1^H and ^31^P MRS profit from the higher SNR and improved spectral dispersion at 7 T, but optimized radiofrequency (RF) hardware is needed to capitalize on these benefits [10]. There are various ways of realizing multinuclear RF coils, falling into two main approaches: either the same physical structure is used for both frequencies, through a frequency splitting trap or by PIN diode switching, or separate structures for the two nuclei are integrated into one coil housing [11]. The single‐structure approach has the advantages of a simpler mechanical setup and fewer elements between which coupling could occur. However, the additional losses of the double‐tuning circuits mean 25%–35% lower SNR compared to single frequency operation [12, 13]; and for PIN diode switching, the transmit power is limited for one of the frequencies by the reverse breakdown voltage of the diode [14, 15]. Tradeoffs between the two frequencies are possible in this approach, making it useful where some ^1^H performance can be sacrificed in favor of the X‐nucleus, and a simpler setup is desired. However, for applications such as the one presented here, strong coil performance is needed for both nuclei. A multi‐structure approach is therefore favorable, as it offers more degrees of freedom for the design and smaller performance loss, if coupling between the structures is mitigated sufficiently.

Coils used for ^31^P/^1^H calf muscle studies commonly comprise one channel per nucleus, realized either as two concentric loops [16, 17] or as double‐tuned or nested birdcage coils. Due to the lower gyromagnetic ratio and corresponding lower Larmor frequency of ^31^P, these approaches work well and continue to be used at both 3 T [18, 19], and 7 T [20]. The concentric loop layout has the advantage of higher sensitivity due to reduced sample noise, at the cost of a small field of view and strong variation of B_1_^+^ with distance from the coil. A birdcage provides lower SNR but more homogeneous transmission and a larger field of view. Commercial implementations of both these designs are available, for example from RAPID (RAPID Biomedical GmbH, Rimpar, Germany), whose multi‐use dual‐tuned flex surface coil for 7 T combines two concentric loops, one for ^1^H, one for ^31^P, and the calf is positioned flat on the coil. RAPID also offers a dual‐tuned extremity coil for 7 T specifically for the calf, which is a volume coil with an inner housing diameter of 22 cm and one channel per nucleus. The cylindrical coil shape allows the calf to be studied in a more natural, rounded conformation.

We have previously reported the design and implementation of a more advanced, multi‐channel setup [21], realized in a three‐channel ^31^P, two‐channel ^1^H calf coil. Different layouts for the ^31^P part of the coil were investigated in simulations, and a half‐cylindrical array of three transceive loops wrapped around the calf was chosen for the final design. This layout outperformed a full birdcage in simulations, with more than 2‐fold SNR gain and higher B_1_^+^ per input voltage. The ^1^H part of the coil consists of two transceive loops layered around the ^31^P array. The offset between the elements provides geometric decoupling between the two arrays.

While this coil has successfully been used in several studies using both ^31^P [22, 23, 24, 25, 26, 27] and ^1^H [3, 28, 29] MRS, optimization of the ^1^H part was not a focus during its development, as it was primarily intended for localization and shimming. SNR limitations can be partly compensated in MRS by using larger voxel sizes and/or averaging, but not without drawbacks. Large volumes may cause partial volume effects and the spectra suffer from increased linewidth. Transmit field homogeneity is also a limiting factor, especially, since some MRS methods, such as double‐quantum filtering for lactate detection, consist of long pulse trains which cannot be made fully adiabatic and are hence particularly sensitive to B_1_^+^ inhomogeneities [30].

The goal of this study was to develop a new ^1^H/^31^P calf coil that provides very high SNR for both ^1^H and ^31^P, to improve the study of metabolites with low natural abundance and in some cases restricted spectral visibility. Additionally, we targeted a strong and homogeneous ^1^H transmit field despite the shortened wavelength at 7 T and parallel imaging capabilities, which will be beneficial for applications such as DTI [31], ASL and other methods requiring good B_1_^+^ homogeneity and parallel imaging for accelerated acquisition in future studies.

Recently [32] we proposed and implemented a combination of three dipoles and six receive‐only loops to serve as the ^1^H part of this new coil. This gave highly efficient and homogeneous ^1^H transmission in the target region of interest (ROI) of gastrocnemius and soleus muscles. It outperformed both our previous ^31^P/^1^H calf coil and a commercial ^1^H‐only knee coil (QED Knee Coil, distributed via Siemens Healthcare, Erlangen, Germany) in terms of SNR, transmit efficiency and homogeneity. It also enabled parallel imaging acceleration factors up to three. In the present work, we extend the previously developed dipole and loop setup by a three‐element ^31^P transceive array [33]. Integrating this into the existing ^1^H setup posed several challenges, mainly with respect to unwanted electromagnetic interactions between the coil elements, which had to be mitigated through several decoupling mechanisms. Additionally, the mechanical complexity of such a high‐density setup with a total of 12 elements had to be addressed. A custom coil housing was developed, including space for the interface components, such as preamplifiers, transmit/receive switches and power dividers. The finished coil's ^1^H and ^31^P performance was tested on phantoms, with the previous ^31^P/^1^H calf coil serving as a reference. The new coil's applicability for in vivo muscle MRS was demonstrated by acquiring an example ^1^H spectrum at rest and time‐resolved interleaved ^1^H/^31^P MRS during a rest‐exercise‐recovery protocol in a single subject.

## 2 Methods

### 2.1 Coil Setup

**FIGURE 1:**
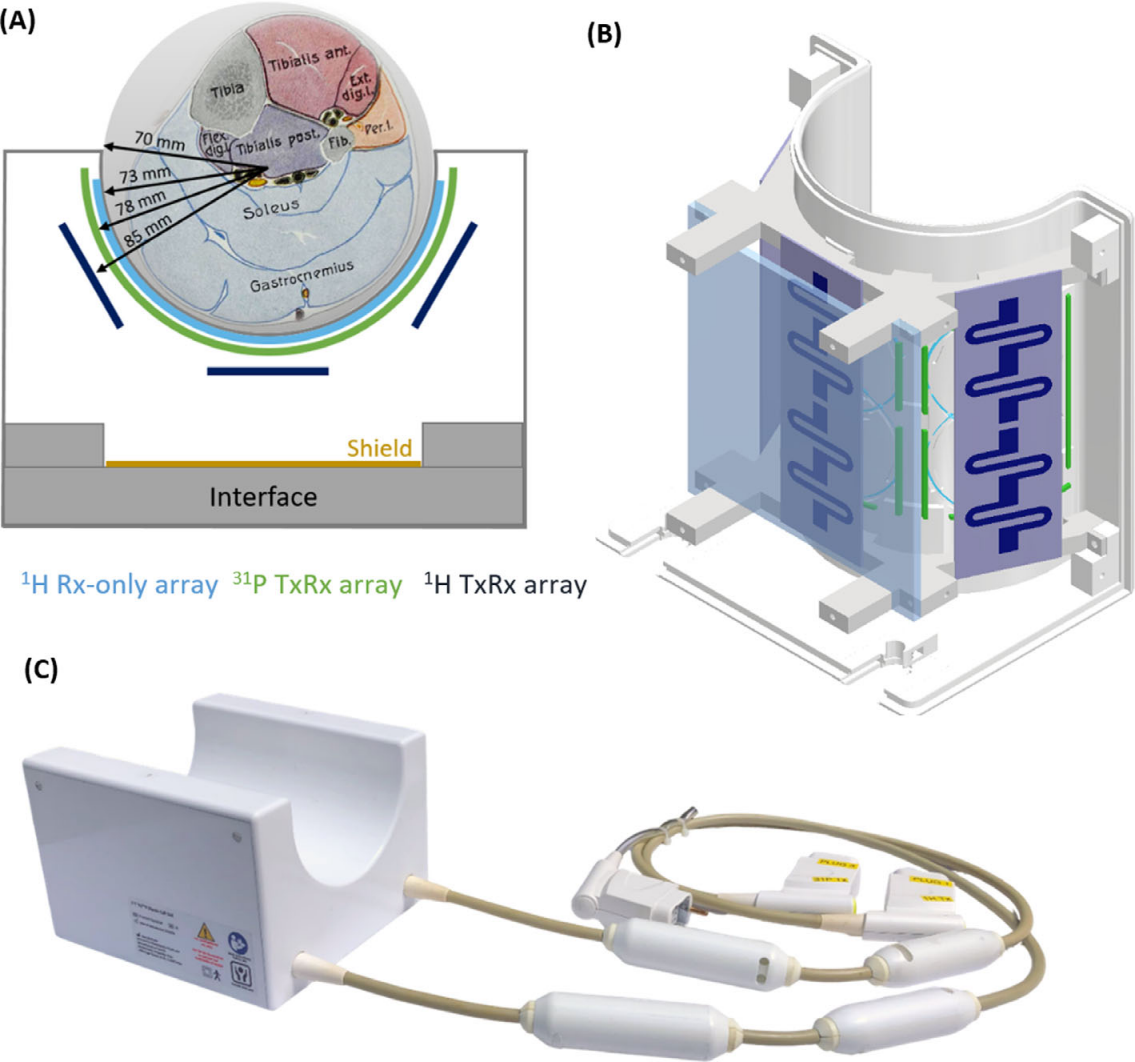
(A) Coil layout in transversal view, with elements color‐coded as noted. (B) Coil elements on base of the housing, covered by transparent interface plate (does not show interface components, shield or lumped elements for tuning and matching). (C) Photograph of the finished coil.

Figure 1 shows the physical arrangements of the new coil. It features three ^1^H transceive dipoles, six ^1^H receive‐only loops and three ^31^P transceive loops, arranged on a half‐cylindrical former with a rectangular base. This is mechanically robust and facilitates subject positioning as the lower leg can be inserted through the open top of the coil and rests comfortably in the former. Furthermore, the design promotes consistent coil loading between subjects, as the calf naturally conforms to the rounded coil surface, improving proximity of the tissue of interest to the coil elements while preserving a near‐natural shape. Inside the housing, the coil elements are arranged in three nested layers wrapped concentrically around the target, gastrocnemius and soleus, the main muscles of the calf.

The outermost layer of coil elements (dark blue in Figure 1A,B) is formed by three ^1^H transceive dipoles, based on a design by Raaijmakers et al. [34], shortened to a length of 150 mm. The dipoles were fabricated as printed circuit boards and mounted at a radial distance of 15 mm from the sample surface using custom holders at either end, which are attached to the former.

To maximize ^1^H SNR, an array of six ^1^H receive‐only loops (light blue in Figure 1A,B) was mounted directly on the former, inset into small grooves which put them only 3 mm from the calf's surface. These loops were fabricated from 1 mm copper wire and are 70 mm in diameter.

Between the two ^1^H arrays, an array of three ^31^P transceive loops (green in Figure 1A,B) was placed 8 mm from the sample surface, with built‐in support structures under the lumped elements at the gaps (see Figure 1B). These rectangular loops were fabricated from 2 mm copper wire and are 115 × 75 mm^2^ in size.

Interface components including preamplifiers, transmit/receive switches and three‐way Wilkinson power dividers for ^1^H and ^31^P were placed at the base of the housing. The latter two were designed and implemented in‐house. The circuit boards were mounted on an acrylic glass plate screwed to the dipole holders connected to the former. This enables flipping the coil upside down for maintenance, directly accessing the interface and the coil elements on the sides after removing the cover; if further access is needed, the interface plate can be removed after unsoldering some cables.

Static *B*_1_^+^ shimming was implemented for both transceive arrays. For the ^1^H dipoles varying the coaxial cable length between the power divider and transmit/receive switches was used to generate the desired phase shift. For the ^31^P array, lumped element phase shifters were used instead of coaxial cables of a specific length, due to the longer wavelength.

Non‐magnetic, high quality factor ceramic capacitors (Exxelia, Paris, France) were used in all coil elements and interface circuits. The larger CPX variant was used for all transmit paths and elements (rated for up to 3.6 kV DC), while the smaller CHB variant was used for the receive‐only elements (up to 1.5 kV DC). To reduce interactions between the dipoles and interface components, a 85 × 150 mm^2^ shield of perforated prototyping board was attached to the bottom of the interface plate; this was found to slightly increase ^1^H B_1_^+^ strength and homogeneity in preliminary experiments.

### 2.2 Decoupling Strategies

**FIGURE 2:**
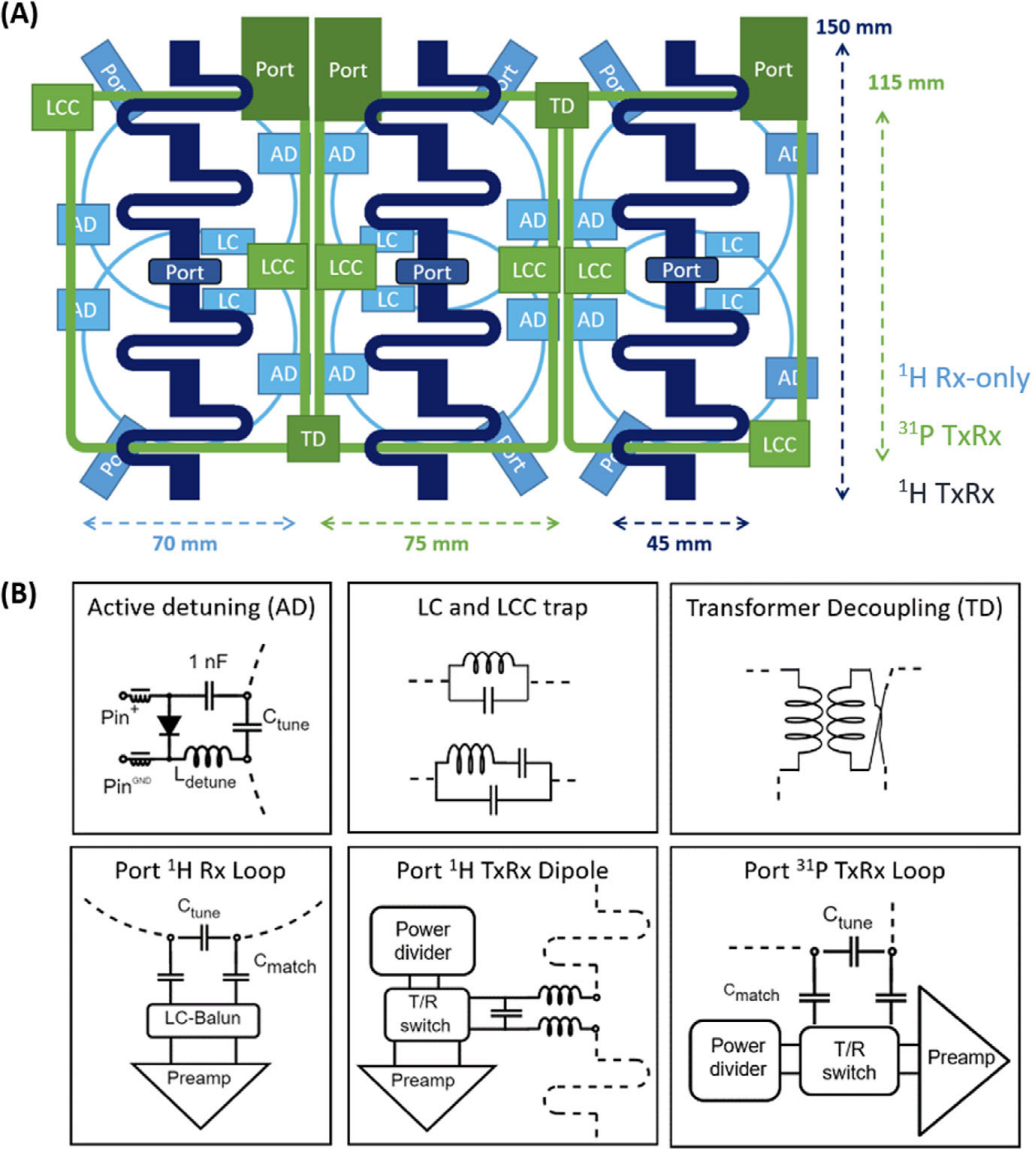
(A) Coil layout in unwrapped view, including the positions of ports, transformer decoupling (TD), active detuning (AD), LC and LCC traps. Tuning capacitors (not shown) were placed in the ^31^P loops at each corner not occupied by an LCC trap. (B) Matching networks and decoupling circuits.

This coil design utilizes multiple decoupling strategies. The coil layout was designed to exploit the intrinsic decoupling of dipoles and loops due to their complementary current patterns [35] (Figure 2A). This enables the simultaneous use of dipoles and loops during reception without excessive noise correlation, resulting in a total of nine ^1^H receive channels with improved sensitivity in depth [32]. It also reduces potential influences of the ^31^P array on ^1^H transmission.

To further reduce interactions, each ^31^P loop includes two LCC traps [36] to block currents at the ^1^H frequency. Neighboring ^31^P loops were transformer decoupled [37]. This allows for easier adjustment than shared capacitor or conductor arrangements, as the decoupling can be optimized by manually changing the inductance and degree of coupling between the two counter‐wound inductors (by either squeezing or stretching). Adjustability in a shared capacitor arrangement would require trimmer capacitors, which are typically not suitable for the high voltages occurring in a transmit array. The inductors for the LCC traps and transformer decoupling were hand‐wound from 1 mm copper wire. The trap inductors were fabricated as toroids, by wrapping 14 turns of the wire around an M6 nylon washer, which corresponds to an inductor diameter of ∼4 mm.

Pairs of ^1^H receive loops under the same dipole were partially overlapped for geometric decoupling, and preamplifier decoupling was established by setting the cable length between the ^1^H receive elements and preamplifiers to provide the desired phase shift. Two active detuning traps were included in each loop for transmission decoupling, one on either side of the dipole, which has been shown to improve transmit efficiency and homogeneity [38]. Each loop also includes a fuse (315 mA current rating), which serves as an additional safety feature and is required by the scanner manufacturer's handbook and EN 60601‐1. LC traps with a ∼400 nH inductor were used to block current at the ^31^P frequency on the ^1^H receive loops, after such coupling was encountered during ^31^P transmit in initial phantom tests. Matching networks and decoupling circuit diagrams are shown in Figure 2B.

### 2.3 Bench Measurements

Tuning, matching, and inter‐element coupling were evaluated by measuring reflection and transmission S‐parameters on a vector network analyzer (E5071C, Keysight Technologies, Santa Rosa, USA). A cylindrical gel phantom (*d* = 140 mm, *l* = 200 mm) manufactured according to the ASTM F2182‐11a standard [39] was used to load the coil. It contained deionized water, 30 mM KH_2_PO_4_, 10 g/L methacrylic acid to reduce convection, and 1 mL/L Gd‐based contrast agent. At 120.3 and 297.2 MHz, the relative permittivity was 80.1 and 75, and conductivity was 0.36 and 0.59 S/m, respectively [21]. Preamplifier decoupling and active detuning of the ^1^H receive loops were evaluated in an unloaded condition using the double‐loop probe method [40]. The LCC traps were tuned before adding them to the coil circuit by measuring their resonance frequency with a sniffer probe while manually adjusting the inductor. Losses in the transmit path caused by power dividers, transmit‐receive switches and cables, were estimated by measuring transmission S‐parameters on the bench for ^1^H and ^31^P interface components.

### 2.4 Phantom MR Measurements

All MR measurements were performed on a whole‐body Magnetom Terra dot Plus 7 T MR scanner (Siemens Healthcare, Erlangen, Germany). Phantom measurements were used to evaluate the coil's ^1^H and ^31^P performance. The following protocol was executed with the presented coil and repeated with the same sequence parameters and slice positions using the two‐channel ^1^H, three‐channel ^31^P reference coil [21], which features a similar, half‐cylindrical geometry with the same inner diameter but a shorter field of view of 110 mm length.

The ^1^H performance was evaluated by acquiring flip angle maps [41], 2D gradient echo images (*T*_R_ = 40 ms, *T*_E_ = 3.69 ms, FOV = 156 mm × 156 mm, 0.5 mm in‐plane resolution, 3 mm slice thickness) and a noise‐only scan on the homogeneous phantom used in the bench tests. The data were evaluated and displayed for a transversal slice using MATLAB R2018b and R2021a (The MathWorks Inc., Natick, MA, USA). Quantitative values were calculated for a semicircular ROI, approximating the gastrocnemius and soleus muscles in vivo. B_1_^+^ maps were calculated from the flip angle maps based on the reference voltage. To compare amplitude and homogeneity, mean and standard deviation of voxel‐wise B_1_^+^ values were calculated across the ROI. SNR and GRAPPA‐accelerated images were reconstructed offline from the gradient echo images and noise scans using the pseudo‐multiple‐replica method with *n* = 200 replicas [42, 43]. Mean and maximum *g*‐factors were calculated from *g*‐factor maps after smoothing with a 2D Gaussian kernel (2.5 mm standard deviation).

To evaluate the ^31^P performance and compare it to the reference coil, a semi‐LASER sequence [44] (*T*_R_ = 5 s, *T*_E_ = 60 ms, 2500 Hz spectral width, 2 preparation scans) was used to acquire a series of 100 spectra from a cylindrical phantom containing 100 mmol/L K_2_HPO_4_. The spectra were acquired from a 50 × 25 × 50 mm^3^ voxel, positioned at 45°, in a location consistent with the gastrocnemius medialis of the left leg in vivo. SNR was calculated for each scan as the ratio between maximum signal and the standard deviation of 700 points of a noise‐only region after 7 Hz line broadening [45]. The average and standard deviation of SNR over 100 scans was calculated.

### 2.5 Full‐Wave Electromagnetic Simulations

To determine the optimal phase settings for static B_1_^+^ shimming for both transceiver arrays, the coil was simulated at both frequencies on a phantom model with geometric and dielectric properties matching the one used in bench and ^1^H MR tests. These simulations were also used to evaluate the coil's B_1_^+^ and B_1_^−^ distribution for ^31^P, since MR‐measurement‐based flip angle and SNR maps cannot be obtained with standard methods due to the low concentration of ^31^P. For safety validation, the B_1_^+^ and SAR distributions were also simulated on the human voxel model Ella from the Virtual Family [46].

The simulations were performed using full‐wave 3D electromagnetic simulation and circuit co‐simulation [47, 48]. The coil elements were modeled in XFdtd (Remcom, State College, PA, USA), with all lumped elements replaced by 50 Ω voltage sources. This enabled an efficient calculation of electric and magnetic fields via the finite‐difference time‐domain (FDTD) method. The lumped elements, including tuning and matching capacitors and decoupling structures were modeled and optimized in Advanced Design System (Keysight Technologies, Santa Rosa, California, USA), with small series resistances to approximate resistive losses in conductors, lumped components, and solder joints.

The scaling factors derived from the circuit co‐simulation were applied to the simulated electromagnetic fields in MATLAB R2021a using a dedicated in‐house toolbox (SimOpTx, Center for Medical Physics and Biomedical Engineering, Medical University of Vienna, Austria). The same toolbox was used to evaluate B_1_^+^, B_1_^−^ and SAR per 10 g of tissue [49, 50]. The phase shifts were optimized in 10‐degree steps by calculating the mean B_1_^+^ and maximum 10 g SAR per unit power for each combination and visually comparing the field homogeneity between the quantitatively most favorable options. Losses in the interface were included by scaling the input power, based on the losses estimated during bench measurements. SAR limits were calculated based on the simulated SAR on the human voxel model, with a safety factor of 1.5 to account for potential simulation inaccuracies and anatomical differences.

### 2.6 In Vivo MRI and MRS

**FIGURE 3:**
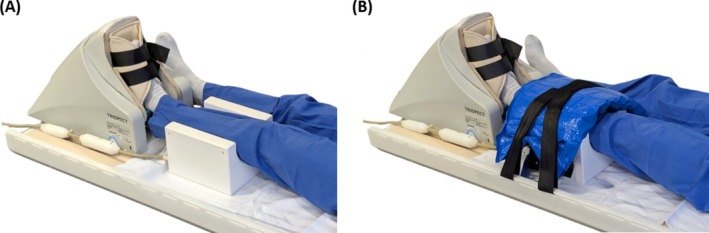
(A) Subject positioning inside the coil for the dynamic exercise studies with an MR‐compatible ergometer. (B) A vacuum cushion and straps were used to minimize unwanted subject movement.

After completing the process of certification for in‐vivo use by the institutional review board and government authority, SNR and B_1_^+^ maps were measured on a healthy subject (male, age 34 years). Example spectra were acquired from the same subject to demonstrate an application of the coil in vivo. After written informed consent was obtained, the subject was positioned feet‐first, supine with his left calf resting in the coil and the left foot positioned inside an MR‐compatible ergometer (Trispect Module, Ergospect GmbH, Innsbruck, Austria), as shown in Figure 3A. To immobilize the leg and coil, a vacuum cushion (MedVac VMR431X01, Kohlbrat & Bunz GmbH, Radstadt, Austria) was placed on the calf at the open sideof the coil, and fixed with belts provided with the ergometer (Figure 3B).

The SNR and B_1_^+^ maps were measured following the methods and sequence parameters described in section 2.4, but *T*_E_ was increased to 3.78 ms for the gradient echo. A water‐suppressed ^1^H spectrum was acquired from the resting gastrocnemius medialis muscle using STEAM [51, 52] with a 10 × 10 × 15 mm^3^ voxel, and *T*_R_ = 3 s, *T*_E_ = 5.68 ms, 32 averages and VAPOR water suppression. Then an interleaved ^1^H/^31^P MRS measurement was performed during a single rest‐exercise‐recovery protocol consisting of 3 min rest, 5 min plantar flexion pushes against the ergometer and 10 min recovery. Spectra were acquired with *T*_R_ = 6 s. To perform the exercise, the subject was instructed to perform two pushes between acquisitions, with the scanner noises serving as timing cues. DRESS [53] was used to acquire the ^31^P spectra with a 2.8 ms sinc pulse and 3.5 ms acquisition delay, from a 15 mm slab in the left gastrocnemius medialis muscle, consistent with [29]. ^1^H spectra were acquired with a semi‐LASER sequence [44], with a 2.8 ms, 7‐lobe sinc pulse for excitation, 180° smoothed chirp adiabatic refocussing pulse, *T*_E_ = 52.8 ms, and a 15 × 15 × 25 mm^3^ voxel. Both sequences used 90° flip angle for excitation, vector size 2048 points, and 3 kHz acquisition bandwidth. All individual spectra were phased and channel‐combined with SNR weighting using an in‐house Python script. Quantification was performed in jMRUI using AMARES [54] to fit lipids, creatine CH_3_, TMA and creatine CH_2_ (Cr2) in the ^1^H spectra, and PCr and Pi in the ^31^P spectra. For the rest‐exercise‐recovery protocol the fitted PCr and Cr2 data were plotted as time courses without averaging. Depletion and recovery time constants were fitted for exercise and recovery, respectively.

## 3 Results

**FIGURE 4:**
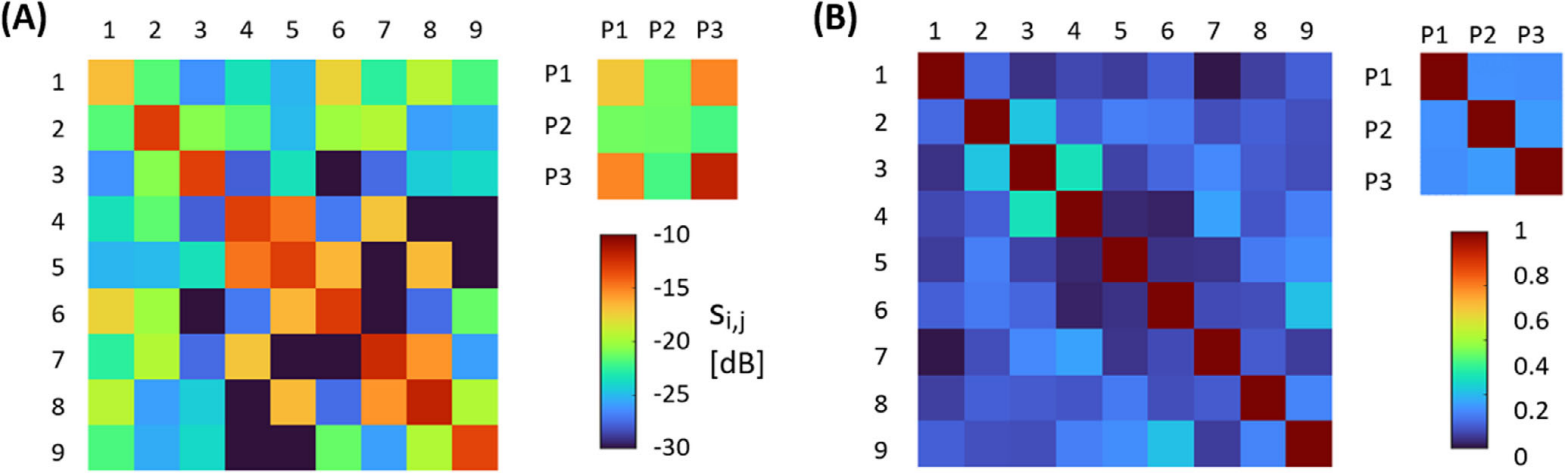
(A) S‐parameter and (B) noise correlation matrix for the ^1^H channels 1–9, and ^31^P channels P1‐P3, measured on the phantoms used for the respective SNR measurements. The channels were numbered left to right when looking into the scanner, starting with the three dipoles followed by the loops closer to the plugs on the scanner bed.

All coil elements were matched to ≤ −12 dB. The maximum coupling between two elements was −14 dB, which occurred between two neighboring, non‐overlapped receive loops. The transformer decoupling between the ^31^P loops resulted in approximately −18 dB coupling, with −15 dB coupling between the outer two loops. The maximum noise correlation between two channels was 33% for ^1^H and 18% for ^31^P. The full S‐parameter and noise correlation matrices are shown in Figure 4. The relative phase shifts for static *B*_1_^+^ shimming were optimized as [0° 120° 240°] for the ^1^H dipole array and [0° 120° 180°] for the ^31^P loop array.

**FIGURE 5:**
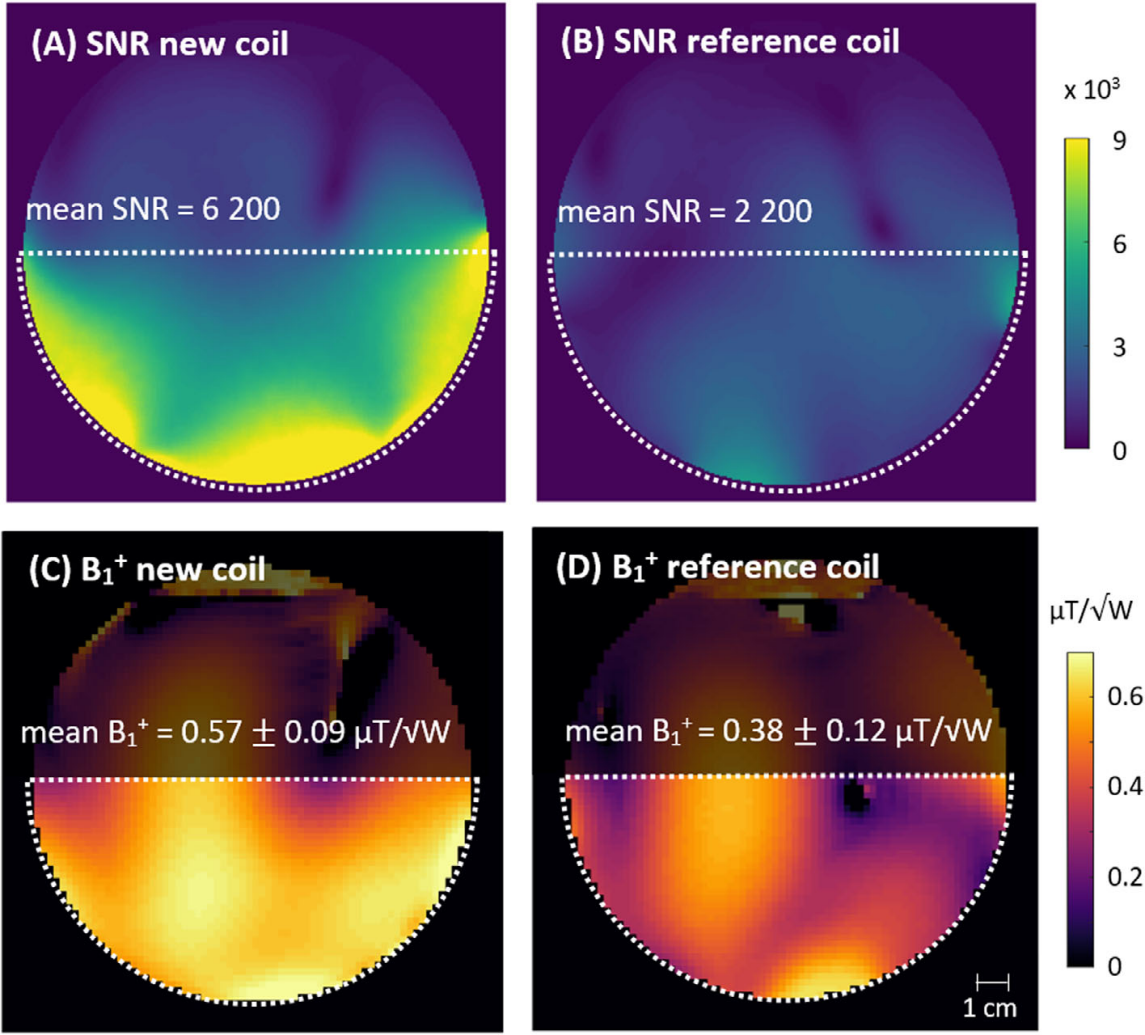
(A, B) Phantom ^1^H SNR and (C, D) B_1_^+^ maps for a transversal slice, comparing the new set‐up (top) to the ^31^P/^1^H reference coil (bottom). The SNR maps were smoothed with a Gaussian kernel (SD = 2.5 mm). Mean values and standard deviations were calculated for the outlined ROI.

**FIGURE 6:**
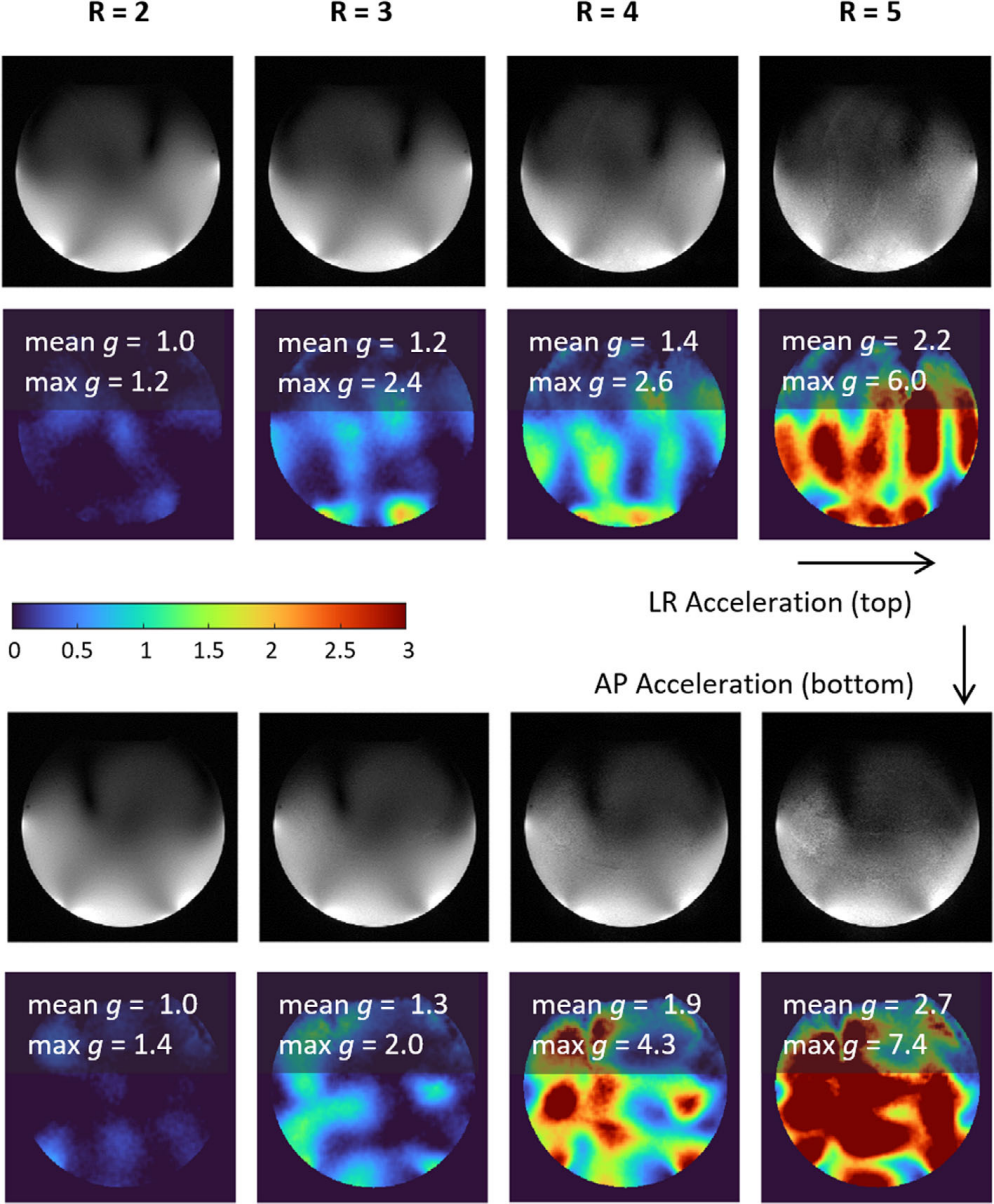
Parallel imaging performance on a homogenous phantom, including 2D gradient echo images (top) and *g*‐factor maps (bottom, with color code), for GRAPPA acceleration by factors *R* = 2, 3, 4, 5 in the LR (upper half) and AP (lower half) directions. Mean *g*‐factors are < 2 for accelerations up to a factor of 4.

Figure 5 shows the ^1^H transmit and receive performance, as SNR and B_1_^+^ maps on the homogeneous phantom. For a semi‐circular ROI representing the gastrocnemius and soleus muscles, the new coil achieved 2.8‐fold higher ^1^H SNR compared to the reference. Its transmit field was stronger and more homogeneous than the reference's, which is reflected in a higher mean value and lower standard deviation of B_1_^+^. The accelerated images and *g*‐factor maps in Figure 6 show that the developed coil enables 4‐fold GRAPPA acceleration in LR and AP direction, with mean *g*‐factors < 2 and only minor fold‐in artifacts.

**FIGURE 7:**
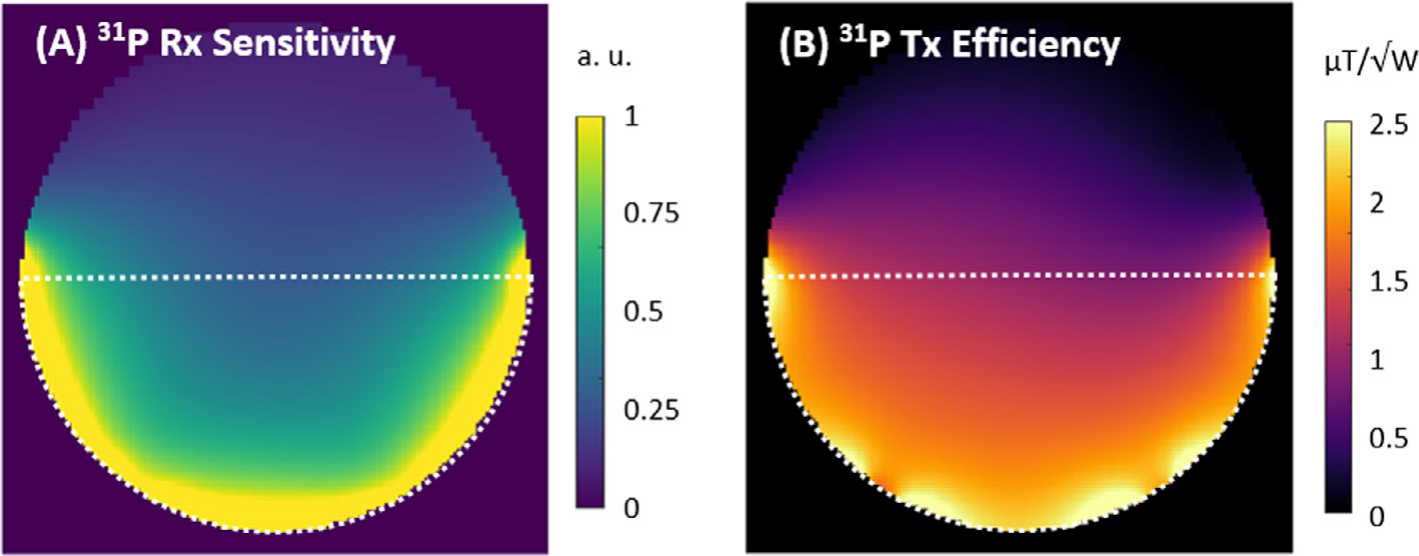
(A) Simulated ^31^P receive sensitivity and (B) transmit efficiency for a central, transversal slice on a homogeneous phantom, with electromagnetic properties matching the gel phantom used for ^1^H SNR and B_1_^+^ measurements.

Simulated ^31^P B_1_^−^ and B_1_^+^ maps of the presented coil on a homogeneous phantom are shown in Figure 7. In measurements on a phantom with 100 mmol/L K_2_HPO_4_, spectral ^31^P SNR was 471 ± 22 for the new coil and 641 ± 26 for the reference, that is, 27% lower with the new coil. For this voxel position the two coils required 200 and 170 V reference voltage, respectively, which is nominally defined as achieving 180° with a 1 ms block pulse.

**FIGURE 8:**
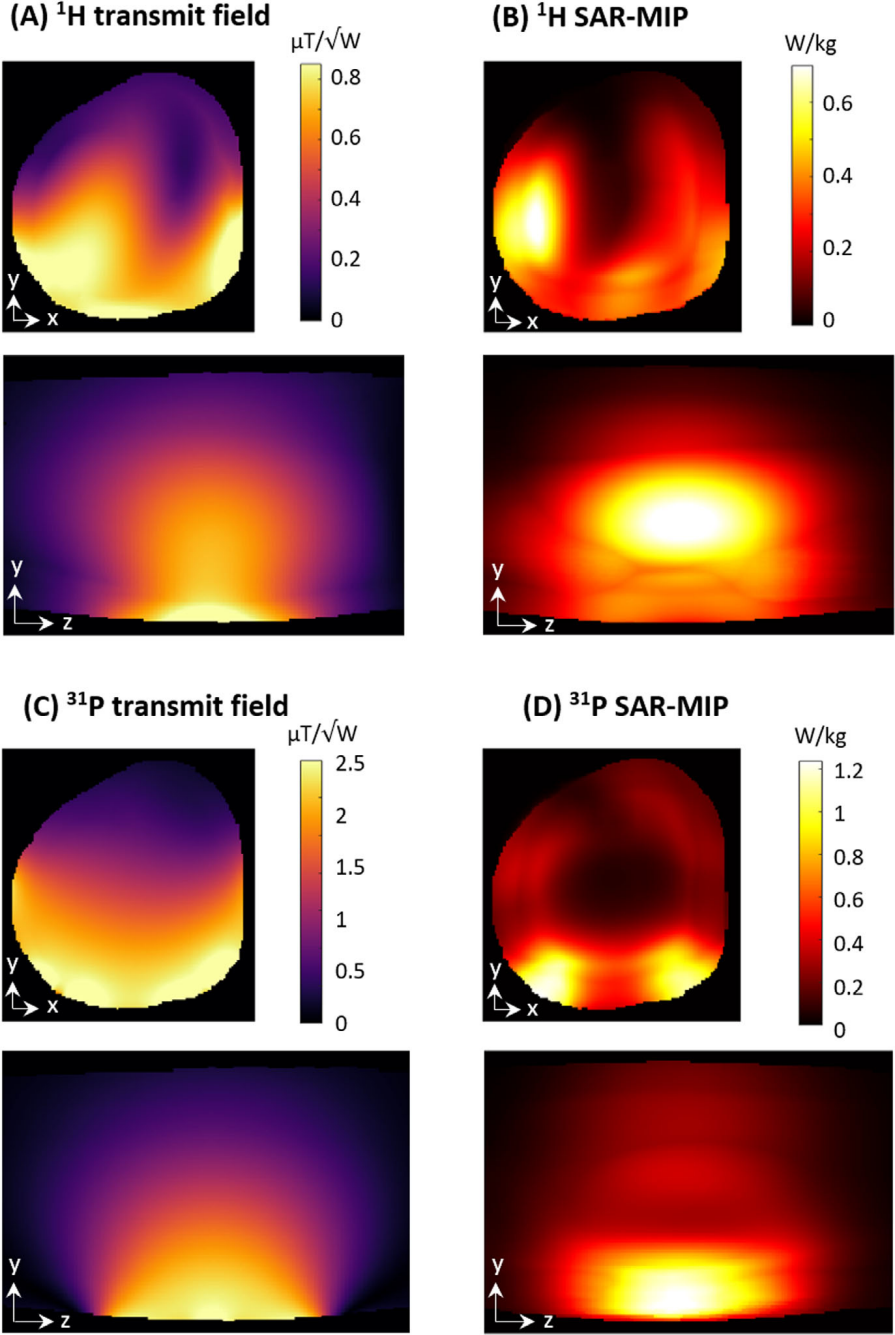
(A, C) Simulated B_1_^+^ maps and (B, D) SAR‐MIPs (maximum intensity projections) for ^1^H (top) and ^31^P (bottom) on central transversal and sagittal slices of an anatomical human voxel model. The data were normalized to input power.

**FIGURE 9:**
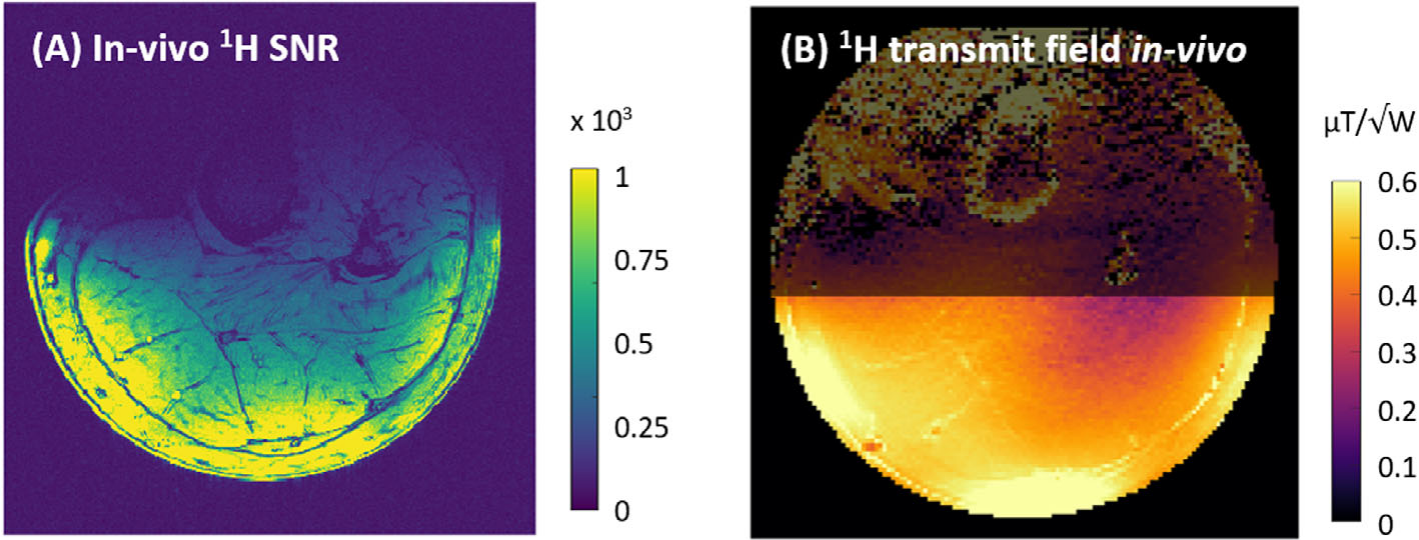
(A) In‐vivo ^1^H SNR map and (B) B_1_^+^ map of a transversal slice across a healthy subject's left calf. The maps were calculated from noise scans, gradient‐echo images and flip‐angle maps.

**FIGURE 10:**
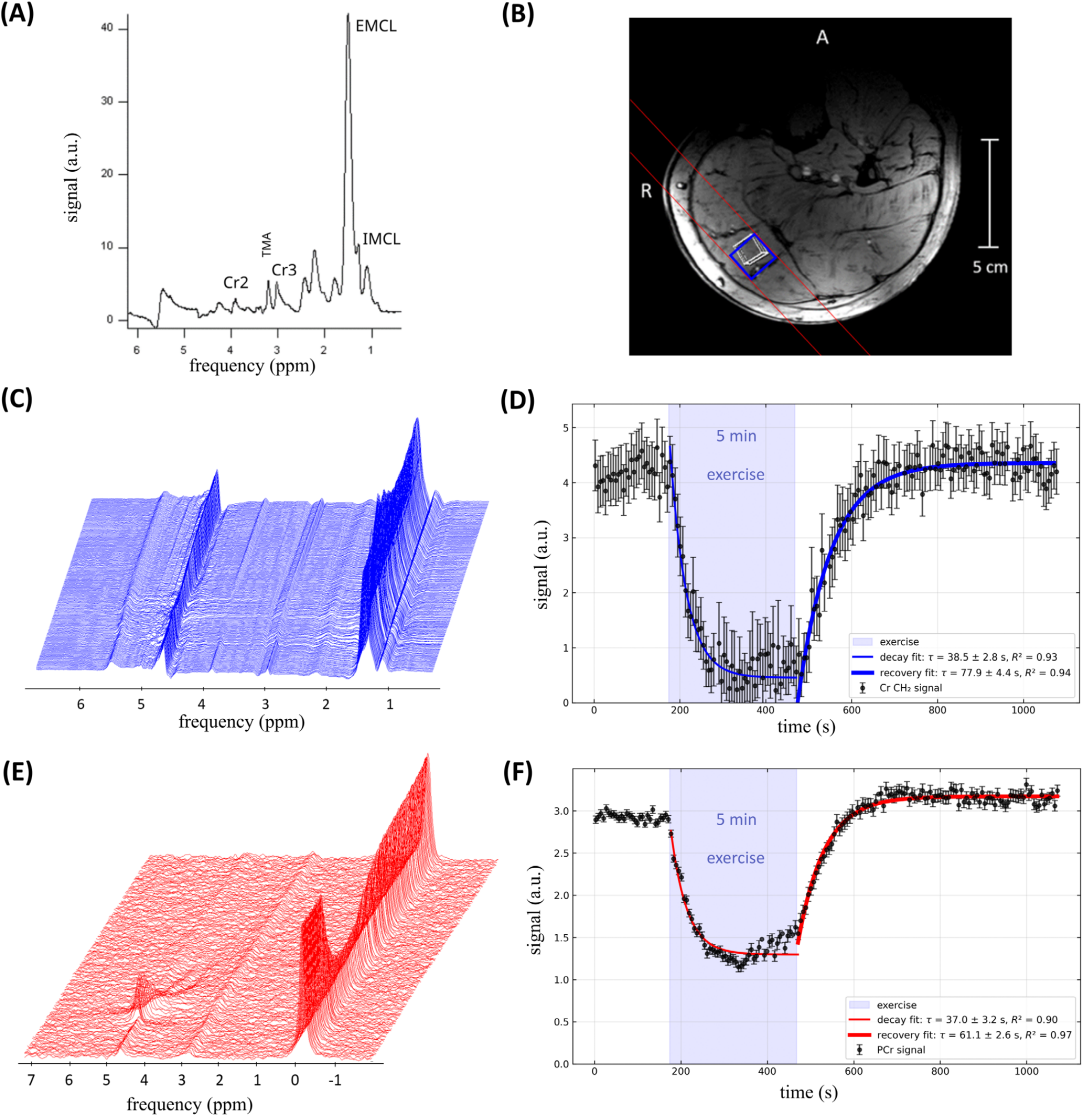
In‐vivo MRS results. (A) Example of water‐suppressed ^1^H STEAM spectrum at rest with 32 averages. (B) voxel positions for the data shown in A, C and E in the gastrocnemius medialis of a healthy subject's left calf. (C), (E) ^1^H and ^31^P spectra acquired during a single exercise bout without averaging. (D), (F) Time courses of Cr2 (top) and PCr (bottom) fitted from data in (C) and (E). The error bars indicate standard deviation of spectral fit. Depletion and recovery time constants were determined with an exponential fit.

Figure 8 shows simulated ^1^H and ^31^P transmit fields and SAR distributions on a human voxel model. The maximum 10 g SAR was 1.2 W/kg for ^31^P and 0.75 W/kg for ^1^H per 1 W of total input power. In‐vivo SNR and B_1_^+^ maps are shown in Figure 9. MRS results from a healthy volunteer and the corresponding voxel positions in the gastrocnemius medialis muscle are shown in Figure 10. This includes an example resting ^1^H spectrum, ^1^H and ^31^P spectra acquired during rest, exercise and recovery and the time courses of fitted PCr and Cr2, showing depletion of both metabolites during exercise and recovery afterwards. The time constants of Cr2 and PCr depletion were *τ*_Cr2‐d_ = 38.5 ± 2.8 s and *τ*_PCr‐d_ = 37.0 ± 3.2 s, and the recovery time constants were *τ*_Cr2‐*r*_ = 77.9 ± 4.4 s, *τ*_PCr‐r_ = 61.1 ± 2.6 s. Comparing amplitudes at the end of exercise and 10 min post‐exercise (to eliminate bias due to possible movement‐related signal change) showed a whole‐exercise depletion of ∼50% for PCr and ∼90% for Cr2.

## 4 Discussion

The phantom ^1^H SNR and B_1_^+^ maps show that the new coil exhibits a substantial performance gain over the reference, with almost three times the ^1^H SNR and an improved ^1^H transmit field amplitude and homogeneity. Furthermore, the coil enables GRAPPA accelerations up to a factor of four, unlike the reference coil, which does not allow any acceleration without severe fold‐over artifacts [32]. Comparing these results to the previously reported ^1^H only version [32], which achieved four‐times higher SNR than the ^31^P/^1^H reference coil, suggests 25% lower ^1^H SNR after adding the ^31^P array. The coil's transmit efficiency and homogeneity were not affected by the addition of the ^31^P array. Slightly lower g‐factors were found for the ^1^H/^31^P coil compared to the ^1^H only version.

The difference in SNR can primarily be attributed to residual coupling with the ^31^P array. Limitations in the comparability of the measurements include differences in sequence parameters (shorter *T*_R_ and *T*_E_ and higher resolution than in [32]) and in the physical implementation of the ^1^H receive loops, which dominate the coil's receive performance. In the ^1^H only prototype version, these were mounted on a thin, flat former, which resulted in closer contact and likely higher SNR than the more robust former required for safe in‐vivo use. Additionally, fuses are required for safe in‐vivo use but add losses and were not included in the preliminary ^1^H only version.

The optimized, relative phase shifts for static B_1_^+^ shimming differ slightly between the ^1^H and ^31^P transceiver arrays. This is partly due to the differing complex B_1_^+^ fields of dipoles and loops. In addition, the port positions for the ^31^P elements are not centered and alternate between the left and right corner of the loop, see Figure 2.

The ^31^P sensitivity of the new coil is 27% lower than the reference coil's. This results from a combination of design differences: While both coils feature similar layout with three transceive loops for the ^31^P part, the element size and sample distance are notably larger for the new coil, with 115 × 75 mm^2^ and 8 mm, compared to 100 × 64 mm^2^ and 4.5 mm. The element length was deliberately increased to extend the field of view, while the distance and width were required to accommodate the ^1^H receive array underneath the ^31^P elements. Some minor losses may also be attributed to resistive components in the LCC traps, which were not used in the reference coil, as well as the aforementioned residual coupling between the concentric ^31^P transceive and ^1^H receive‐only loops.

This coil is intended for ^1^H MRS of metabolites that are severely limited in terms of SNR and detectability, such as the small CH_2_ resonance of creatine or lactate, which requires the suppression of lipids at the same resonance frequency. ^1^H performance was therefore prioritized over ^31^P SNR, which in previous studies using the reference coil was not a limiting factor [22, 26, 27, 29]. This design choice is supported by the results in Figure 10, which show a typical application of this coil, where ^31^P SNR still exceeds the SNR of ^1^H MRS.

The in‐vivo ^1^H SNR and B_1_^+^ maps in Figure 7 show a strong, homogeneous B_1_^+^ and high SNR, particularly in the gastrocnemius medialis muscle, which is one of the most common targets for dynamic exercise studies. The measured B_1_^+^ distribution corresponds well to the simulated fields on a human voxel model (Figure 8). The lower amplitude in the measurements suggests that in addition to some anatomical differences between the voxel model and subject, some losses may have been underestimated in the simulation. Since a safety factor is applied when calculating SAR limits from the simulated data, minor deviations will not affect patient safety.

The example ^1^H STEAM spectrum in Figure 10A demonstrates the coil's ability to acquire high‐quality spectra from small voxels, with clearly showing the creatine CH_2_ peak and clear distinction between TMA, Cr CH_3_ and intra and extra‐cellular lipid peaks. The small Cr2 peak is also distinguishable in the un‐averaged ^1^H spectra during exercise and could be fitted with consistent certainty, even at 90% depletion at the end of the exercise portion of the protocol (Figure 10D). The Cr2 depletion and recovery time constants correspond well to those fitted from the PCr data (Figure 10F). The difference in relative depletion of 50% for PCr and 90% for Cr2 could be explained at least partly by the larger volume of the DRESS slab used for the ^31^P acquisition, which includes other regions of the muscle than the semi‐LASER voxel used for ^1^H acquisition. Also, the exact mechanism of altered Cr2 visibility [4] is still not fully elucidated [29, 55].

Interleaving multi‐nuclear acquisitions has the advantage of acquiring two datasets in a single scan session, which reduces measurement time and, more importantly, allows multiparametric information to be obtained from the same physiologic state, which could not be reproduced exactly in consecutive measurements [56]. ^1^H SNR can be increased further in such measurements by acquiring several ^1^H spectra for each ^31^P acquisition [56, 57] (within the constraints of SAR, *T*_1_ saturation effects and the time required for exercising). The focus of this work is on the coil development and quantitative performance assessment, with the in‐vivo data primarily intended to demonstrate its applicability. Future work will include a more detailed investigation of the coil's performance on multiple subjects with different calf anatomies.

The new coil design successfully integrates multiple different decoupling strategies, ranging from geometric decoupling enabled by the complementary current patterns of loops and dipoles, to decoupling circuits such as transformer decoupling, LC and LCC traps. This results in a high‐density, multinuclear array that combines nine ^1^H and three ^31^P channels, with limited electromagnetic interactions. The custom coil housing provides mechanical stability for the coil elements, cables and interface components. This enables easy handling and ensures subject safety, which will be particularly valuable for studies in healthy subjects or patients. The coil's excellent ^1^H performance will allow existing MRI and MRS protocols to be performed with improved accuracy and higher temporal or spatial resolution. Its parallel imaging capability will improve muscle fiber tracking using DTI, which in turn will provide valuable information for optimizing sequence parameters for measurements of metabolites with orientation‐dependent visibility like creatine or lactate. The higher ^1^H SNR and improved transmit homogeneity will enable the use of new sequences that were previously limited by insufficient SNR to be implemented in vivo, which will be valuable in generating new methods of studying muscle metabolism.

## Funding

This research was funded in whole or in part by the Austrian Science Fund (FWF) [https://doi.org/10.55776/P35305 to M.M. and 10.55776/I6755 to R.F.]. For open access purposes, the author has applied a CC BY public copyright license to any author accepted manuscript version arising from this submission.

## Data Availability

The data that support the findings of this study are available from the corresponding author upon reasonable request and on Zenodo at 10.5281/zenodo.17986476.
